# Prevalence of *BRCA1* and *BRCA2* pathogenic and likely pathogenic variants in non-selected ovarian carcinoma patients in Brazil

**DOI:** 10.1186/s12885-018-5235-3

**Published:** 2019-01-03

**Authors:** Deborah Porto Cotrim, Adriana Regina Gonçalves Ribeiro, Daniele Paixão, Diogo Cordeiro de Queiroz Soares, Rima Jbili, Natasha Carvalho Pandolfi, Camila Cezana, Carine de Cássia Mauro, Henrique Mantoan, Graziele Bovolim, Louise de Brot, Giovana Tardin Torrezan, Dirce Maria Carraro, Glauco Baiocchi, Maria Nirvana da Cruz Formiga, Alexandre A. B. A. da Costa

**Affiliations:** 1 Department of Medical Oncology, AC Camargo Cancer Center, Rua Professor Antonio Prudente 211, São Paulo, CEP: 01509-900 Brazil; 2 Department of Oncogenetics, AC Camargo Cancer Center, Rua Professor Antonio Prudente 211, São Paulo, CEP: 01509-900 Brazil; 3 Department of Gynecologic Oncology, AC Camargo Cancer Center, São Paulo, Brazil; 4 Department of Pathology, AC Camargo Cancer Center, São Paulo, Brazil; 5 Genomics and Molecular Biology Laboratory, AC Camargo Cancer Center, São Paulo, Brazil

**Keywords:** Ovarian cancer, *BRCA1* and *BRCA2* germline mutations, Brazil, Prevalence

## Abstract

**Background:**

*BRCA1/2* pathogenic (P) and likely pathogenic (LP) germline variants are frequent among patients with ovarian carcinoma. However, these variants have not been extensively characterized in patients with ovarian cancer in Brazil.

**Methods:**

In this retrospective study we evaluated clinical characteristics and *BRCA1/2* genetic test results from patients with ovarian carcinoma who underwent genetic counseling at A.C.Camargo Cancer Center (Brazil) between 2015 and 2017 and had performed germline genetic testing of *BRCA1/2* genes.

**Results:**

Among 158 patients, 33 P and LP variants and were found (20.8%), 27 in *BRCA1* and six in *BRCA2,* and six variants of unknown clinical significance (VUS). Thirteen percent of the patients did not have Multiplex Ligation-dependent Probe Amplification (MLPA) results. Three P variants in *BRCA1* were found in more than one patient: c.5266dupC (p.Gln1756Profs*74), c.3331_3334delCAAG (p.Gln1111Asnfs5*), and c.211A > G (p.Arg71Gly). One LP variant in *BRCA1* had not been previously described, c.4153_4154delCT (p.Leu1385Ilefs*5). Patients with previous diagnosis of breast cancer were carriers of P or LP variant in 8 of 12 cases (66.7%), and patients with a family history of ovarian or breast cancer in first- or second-degree relatives were carriers of P or LP variant in 26.7% of cases compared to 16.9% for patients without family history (*p* = 0.166).

**Conclusion:**

Prevalence of *BRCA1/2* germline P and LP variants is slightly higher than previously described by the largest occidental studies, with a high prevalence of variant c.5266dupC (p.Gln1756Profs*74) in *BRCA1* observed. Moreover, we identified a new LP variant.

## Background

Ovarian cancer is the most lethal gynecological cancer. In the United States, 22,240 new cases and 14,070 deaths due to ovarian cancer are expected, making it as the 5th most lethal cancer among women [1]. In Brazil, 6150 new cases are expected in 2018 [2]. The high lethality is partially due to diagnosis of the disease in its advanced disease stages in most cases. Even if screening can lead to small benefits in selected high risk groups [3, 4] there is still an unmet need for early diagnosis strategies.

Hereditary Breast and Ovarian Cancer Syndrome (HBOC) occur most often in the presence of germline *BRCA1* or *BRCA2* pathogenic variants. Carriers of *BRCA1* and *BRCA2* pathogenic variants have a risk of developing ovarian cancer about 45 and 20% until 80 years old, respectively [5]. Risk-reducing salpingo-oophorectomy decreases the incidence and mortality due to ovarian cancer in this high risk group [6]. Moreover, in the last decade the emergence of PARP inhibitors expanded the importance of *BRCA* pathogenic variants detection not only to prevention but also to treatment of ovarian cancer patients [7–9].

Earlier studies evaluating exclusively ovarian cancer patients found a frequency of 11 to 15% germline pathogenic variants in *BRCA1* or *BRCA2* among epithelial ovarian cancer patients [10–15]. The ovarian cancer TCGA confirmed a frequency of 17% germline pathogenic variants in *BRCA1* or *BRCA2* in 489 unselected ovarian cancer cases [16]. More recent studies in populations from different countries showed a wider range of frequency of pathogenic variants in *BRCA1* or *BRCA2* depending on the study, with the highest frequencies found in Asian populations reaching 27% of ovarian cancer patients [17–23].

Germline pathogenic variants in other genes related to homologous recombination pathway have also been associated with hereditary ovarian cancer such as *PALB2, RAD51C* and *RAD51D*, and are found in about 3 to 5% of ovarian cancer patients [18, 24].

To the best of our knowledge there is only one study that did a comprehensive evaluation of *BRCA1* and *BRCA2* variants in 100 ovarian cancer patients in Brazil and found a frequency of 19% of pathogenic variants [25]. On the other hand, a second study evaluating only five specific variants in *BRCA1* and three in *BRCA2* in 103 patients found no pathogenic variants [26].

Since 2014, *BRCA* germline testing has been done routinely after genetic counseling at our institution for epithelial ovarian cancer patients. The aim of the present study is to evaluate the frequency of pathogenic and likely pathogenic variants in *BRCA1* and *BRCA2* among epithelial ovarian cancer Brazilian patients and compare clinical features of *BRCA1/2* carriers and non-carriers.

## Methods

### Patients

**Fig. 1:**
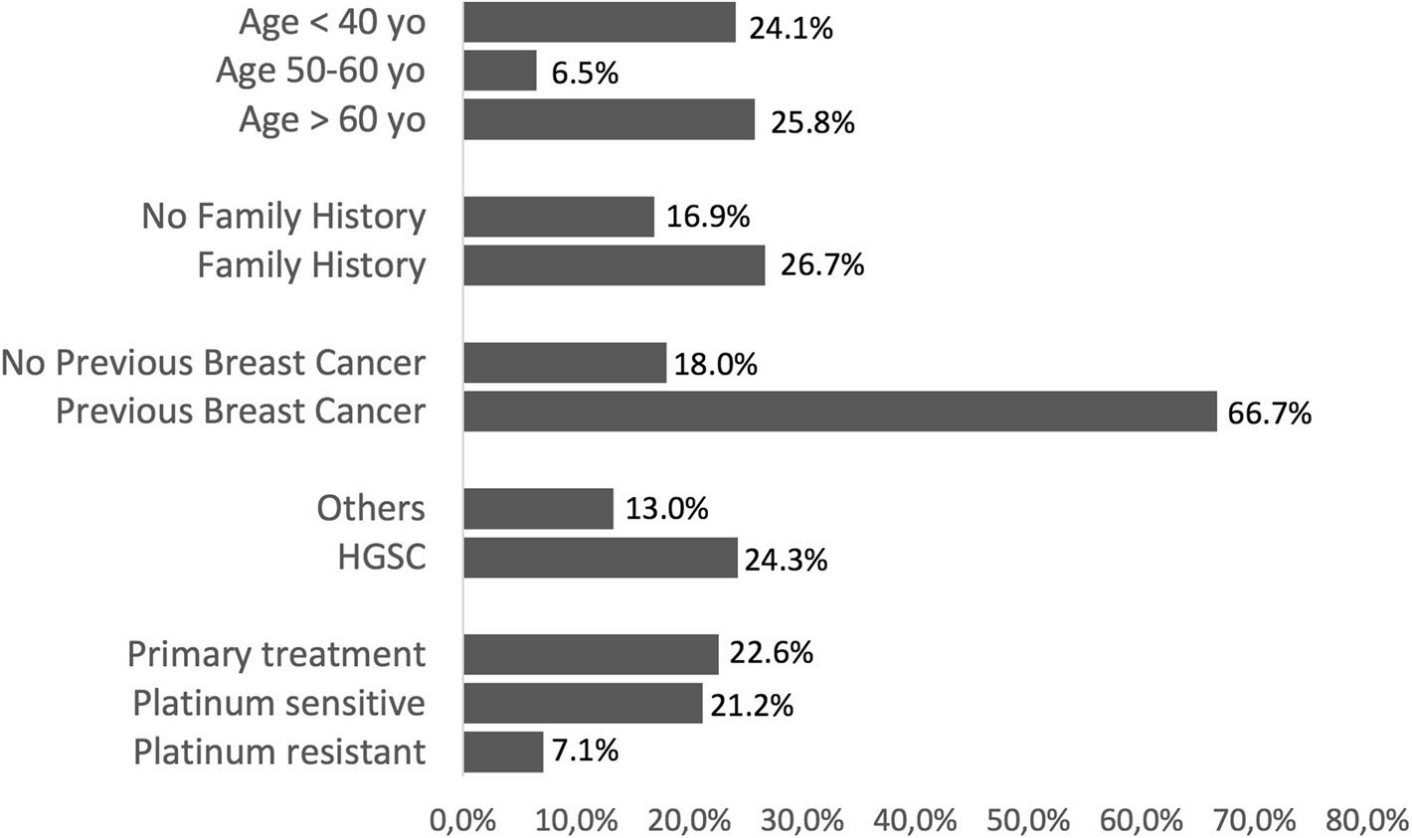
Flowchart representative of the inclusion criteria adopted in the study

It is a retrospective cohort study including all consecutive patients with ovarian carcinoma tested for *BRCA1* and *BRCA2* germline mutations seen for genetic counseling in the Oncogenetics Department at A.C. Camargo Cancer Center from January 2015 to November 2017 irrespective o the date of diagnosis (Fig. 1).

### Genetic test results

All patients included in the study went through genetic counseling consultation at the Oncogenetics Department. Patients were referred to germline *BRCA1* and *BRCA2* genetic testing, which was performed in different commercial laboratories. Data on identified variants were retrieved from genetic test reports. Patients who presented no pathogenic or likely pathogenic variants in *BRCA1* or *BRCA2* were routinely recommended to follow the investigation looking for copy number alterations with MLPA. Patients without MLPA results were not excluded from the study.

All pathogenic and likely pathogenic variants and variants of uncertain significance reported by commercial laboratories were reviewed at the Genomics and Molecular Biology Laboratory at A.C. Camargo Cancer Center and reclassified according to the American College of Medical Genetics (ACMG) guidelines in a five tier classification: 1 = benign (B); 2 = likely benign (LB); 3 = variant of unknown significance (VUS); 4 = likely pathogenic (LP); 5 = pathogenic(P) [27].

### Clinical data

Clinical findings were retrieved from the medical records. Baseline characteristics included date of diagnosis, age at diagnosis of ovarian cancer, tumor histological subtype, staging, personal history of ovarian and breast cancer, pretreatment CA125 level. Family pedigrees were reviewed to identify family history of ovarian or breast cancer.

Data on treatment and follow up from the diagnosis until the last date of consultation at the hospital were also retrieved and included: primary versus interval debulking surgery, date of surgery, residual disease, chemotherapy used at first-line treatment, date of last platinum infusion at first-line treatment, response to chemotherapy, date of first recurrence. Data on recurrences included: treatment with secondary debulking surgery, chemotherapy used, date of first and last chemotherapy infusion, response to chemotherapy, and date of disease progression.

Recurrence was defined according to the GCIG (Gynecological Cancer Intergroup) criteria after the analysis of RECIST (Response Evaluation Criteria in Solid Tumors) and CA125 progression was obtained from the medical records. The date of the earlier event was considered for progression [28, 29]. The recurrence detected after 6 months of the last platinum infusion was defined as platinum sensitive recurrence. The recurrence detected within less than 6 months after the last platinum infusion was defined as platinum resistant recurrence. All recurrences that followed this first platinum resistant recurrence were also considered as platinum resistant. Progression-free survival (PFS) was defined as the interval between the date of diagnosis and disease progression or death by any cause. Overall survival (OS) was defined as the interval between the dates of diagnosis and death by any cause. The interval between the date of the last platinum compound infusion and the date of the disease progression was defined as platinum-free interval (PFI).

Response to platinum retreatment data was retrieved from the medical records, and revised for clinical findings, data of CA125 levels and image reports were also collected. GCIG criteria were used to evaluate RECIST and CA125 response [28, 29]. In accordance, each case was categorized as having “response” (complete or partial response) or “no response” (stable disease or disease progression).

### Statistical analysis

Statistical analyzes were performed using the SPSS (v. 21.0; SPSS, Chicago, IL, USA) software, adopting a two-tailed *P* < 0.05 value as significant.

Frequencies, medians and interquartile range (IQR) were used to describe patients’ characteristics and genetic test findings.

The associations between clinical characteristics and response rate to chemotherapy with presence of pathogenic or likely pathogenic variants were investigated using Qui-square test or Fisher’s Exact test when necessary.

Overall survival and progression free survival analyses were performed using Kaplan-Meier, log rank test, and hazard ratios were calculated with cox regression analysis.

## Results

One hundred and fifty-eight patients were included. Median age was 54.7 years (IQR = 43.1 years to 67.7 years), 70.3% presented high grade serous carcinoma (HGSC), 23.3% presented at FIGO stages I or II and 80.7% presented at FIGO stages III or IV. Baseline clinical characteristics are described in Table 1 according to the final mutational status.

**Table 1:** Clinical and Pathological features of ovarian cancer patients according to pathogenic and likely pathogenic *BRCA1* and *BRCA2* variants

Characteristics	BRCA 1/2 wt (*N* = 125)	BRCA 1/2 mut (*N* = 33)	p
Median age at diagnosis in years (IQR)	54.44 (42.7–67.7)	60.65 (43.8–67.2)	0.946
Time between diagnosis and genetic testing in months (IQR)	23.91 (10.75–45.15)	18.95 (8.86–39.77)	0.390
Histology			0.109
Serous	83 (66.4)	27 (81.8)	
Non-serous	40 (32.0)	6 (18.2)	
Missing	2 (1.2)	0 (0)	
FIGO stage			0.288
I/II	29 (23.2)	5 (15.1)	
III/IV	86 (68.8)	26 (78.8)	
Missing	10 (8.0)	2 (6.1)	
Personal history of breast cancer			0.001
Positive	4 (3.2)	8 (24.2)	
Negative	105 (84.0)	22 (66.6)	
Missing	16 (12.8)	3 (9.1)	
Family history for breast and/or ovarian cancer			0.115
Positive (first and/or second degree relatives)	55 (44.0)	20 (60.6)	
Negative	54 (43.2)	11 (33.3)	
Not completely Known/ Unknown	16 (12.8)	2 (9.1)	
Median pretreatment CA-125 (IQR)	436.5 (117.5–1163.0)	681.5 (126.5–2062.5)	0.351
Treatment			0.414
First-line	65 (52.0)	19 (57.7)	
Platinum-sensitive	41 (32.8)	11 (33.3)	
Platinum-resistant	13 (10.4)	1 (3.0)	
Unknown	6 (4.8)	2 (6.0)	

BRCA1/2 wt = no pathogenic or likely pathogenic variants. BRCA1/2 mut = presence of pathogenic or likely pathogenic variant. *IQR* Interquartile range

### Genetic test results

Median time from diagnosis of ovarian cancer to genetic testing was 21.6 months (IQR 10.3 months to 44.5 months). Most patients (53.2%) were tested after first line treatment and before first recurrence, 32.9% were tested in the platinum sensitive recurrent setting, and 8.9% were tested in the platinum resistant setting. Only 21 patients (13.3%) did not have MLPA results available.

Forty-five variants classified as P, LP or VUS were identified among the 158 tested patients according to commercial laboratories reports. After reviewing and reclassifying all P, LP variants and VUS according to ACMG Guidelines we found conflicting interpretation in 8 variants. In *BRCA1,* the variant c.67_75delGAGTGTCCC (p.Glu23_Pro25del) first classified as VUS was reclassified as LP, based on experimental analysis of three tumors from this patient that showed loss-of-heterozygosity for the normal *BRCA1* allele (data not shown). The variant c.4964C > T (p.Ser1655Phe) first classified as P was reclassified as LP, the variant c.2368A > G (p.Thr790Ala) and c.1067A > G (p.Gln356Arg) both first classified as VUS were reclassified as LB and B respectively. In *BRCA2* the variants c.794-22C > T, c.7601A > G (p.Ala2534Val), c.1792A > G (p.Thr598Ala), and c.4928 T > C (p.Val1643Ala), first classified as VUS were all reclassified as LB.

After reclassification there were 33 P or LP variants, representing a frequency of 20.8%, 27 in *BRCA1* and six in *BRCA2,* and six VUS, three in *BRCA1* and three in *BRCA2.* One of the 33 P or LP variants was a large deletion of exon 16 in *BRCA1*. All variants first classified as P, LP or VUS according to commercial laboratories are described in Table 2 according to their reclassification status.

**Table 2:** Description of *BRCA1* and *BRCA2* variants and clinical and pathological characteristics

ID	HGVS cDNA	HGVS protein	ACMG Classification	Variant Type	Age Ranges	Histology	FIGO stage	Family History^a^
*BRCA1*								
1	c.4153_4154delCT	p.Leu1385llefs^*^5	LP	F	40-49	S	IIIC	+
2	c.5074 + 2 T > C	p.?	P	Ss	60–69	LGSC	IIIC	+
3	c.211A > G	p.Arg71Gly	P	M	40–49	S	IIIC	+
4	C.4484G > T	p.Argt1495Met	P	M	40–49	E	IIIC	+
5	c.1612C > T	p.Gln538Ter	P	N	60–69	S	IV	+
6	c.3331_3334delCAAG	p.Gln1111Asnfs^*^5	P	F	40-49	S	IIIC	+
7	c.3331_3334delCAAG	p.Gln1111Asnfs^*^5	P	F	30-39	S	IIIC	+
8	c.3331_3334delCAAG	p.Gln1111Asnfs^*^5	P	F	50-59	S	IIIC	+
9	c.5266dup	p.Gln1756Profs^*^74	P	F	60-69	S	IV	–
10	c.5266dup	p.Gln1756Profs^*^74	P	F	70-79	S	IV	+
11	c.5266dup	p.Gln1756Profs^*^74	P	F	40-49	S	IIIC	+
12	c.5266dup	p.Gln1756Profs^*^74	P	F	50-59	UC	IIIC	–
13	c.5266dup	p.Gln1756Profs^*^74	P	F	40-49	S	IIIC	+
14	c.1687C > T	p.Gln563Ter	P	N	50–59	S	IV	–
15	c.67_75delGAGTGTCCC	p.Glu23_Pro25del	LP	Indel	60–69	S	IIIC	–
16	c.4117G > T	p.Glu1373Ter	P	N	40–49	UC	IIB	–
17	c.3477_3480delAAAG	p.Ile1159Metfs	P	F	50–59	S	IIIC	+
18	c.188 T > A	p.Leu63Ter	P	N	40–49	S	IV	+
19	c.1961delA	p.Lys654Serfs^*^47	P	F	60-69	S	IIIC	+
20	c.3270_3273delACCT	p.Pro1091Argfs^*^17	LP	F	40-49	S	IIIB	–
21	c.798_799delTT	p.Ser267Lysfs^*^19	P	F	50-59	S	IIIC	+
22	c.2368A > G	p.Thr790Ala	LB	M	30–39	LGSC	IIIC	–
23	c.5558A > G	p.Tyr1853Cys	US	M	50–59	S	IIIC	+
24	c.533 T > C	p.Val178Ala	US	M	30–39	E	IIIC	–
25	c.2077_2078insTA	p.Asp693Valfs^*^9	P	F	40-49	S	IV	+
26	c.1067A > G	p.Gln356Arg	B	M	60–69	S	IIIC	–
27	c.5123C > A	p.Ala1708Glu	P	M	40–49	S	IIIC	+
28	c.1066C > T	p.Gln356Ter	P	N	50–59	S	IV	–
29	c.4964C > T	p.Ser1655Phe	US	M	40–49	E	IIIC	+
30	c. 211A > G	p.Arg71Gly	P	M	40–49	S	IIIC	+
31	del exon 16	p?	P	D	40–49	S	IIIC	–
32	c.547 + 2 T > A	p?	P	Ss	50–59	S	IV	–
*BRCA2*								
33	c.4129A > G	p.Asn1377Asp	US	M	30–39	S	IIIC	–
34	c.8488-1G > A	p.?	P	Ss	50–59	S	IIIC	–
35	c.794-22C > T	p.?	LB	Ss	30–39	S	IIIC	–
36	c.7601C > T	p.Ala2534Val	LB	M	60–69	CC	IV	+
37	c.323A > G	p.Asn108Ser	US	M	70–79	S	IIIC	
38	c.6491_6494delAGTT	p.Gln2164Argfs^*^3	P	F	50–59	CS	IIIC	+
39	c.3680_3681delTG	p.Leu1227Glnfs^*^5	P	F	60-69	S	IIIC	–
40	c.8878C > T	p.Gln2960Ter	P	N	50–59	S	IIIC	+
41	c.6656C > G	p.Ser2219Ter	P	N	50–59	S	IIIC	–
42	c.1792A > G	p.Thr598Ala	LB	M	40–49	S	IC	–
43	c.4928 T > C	p.Val1643Ala	LB	M	80–89	S	IIIC	–
44	c.738delT	p.Phe246Leufs^*^5	LP	F	40-49	S	IV	–
45	c.9101A > G	p.Gln3034Arg	US	M	80–89	S	IV	+

*P* Pathogenic variant, *LP* Likely pathogenic variant, *US* Variant of uncertain significance, *LB* Likely benign variant, *B* Benign variant, *F* Frameshift, *N* Non sense, *M* Missense, *IndDel* Inframe insertions or deletions, *Ss* Splice site, *D* Large deletion, *S* High grade serous carcinoma, *E* Endometrioid carcinoma, *UC* Undifferentiated carcinoma, *CC* Clear cell carcinoma, *CS* Carcinossarcoma, *LGSC* Low grade serous carcinoma

^a^Positive family history if: history of breast or ovarian carcinoma in first or second degree relatives or patient with previous breast cancer

Three variants were found in more than one patient, all in *BRCA1*: the Ashkenazi founder mutation c.5266dupC (p.Gln1756Profs*74) was found in five patients. One of the five patients carrying this variant had a recognized Jewish ancestry; the variant c.3331_3334delCAAG (p.Gln1111Asnfs5*) was found in three patients; and the variant c.211A > G (p.Arg71Gly) was found in two patients.

One LP variant in *BRCA1* has not been described before in Brazilian patients, nor in any consulted public databases (Clinvar, BIC and LOVD) c.4153_4154delCT (p.Leu1385Ilefs*5).

### Pathogenic and likely pathogenic variants and clinical characteristics

Median age at diagnosis and median time from diagnosis to genetic testing was not different between P and LP variant carriers and non-carriers. FIGO stage and CA125 baseline levels were also not different between P and LP variant carriers and non-carriers (Table 1).

**Fig. 2:**
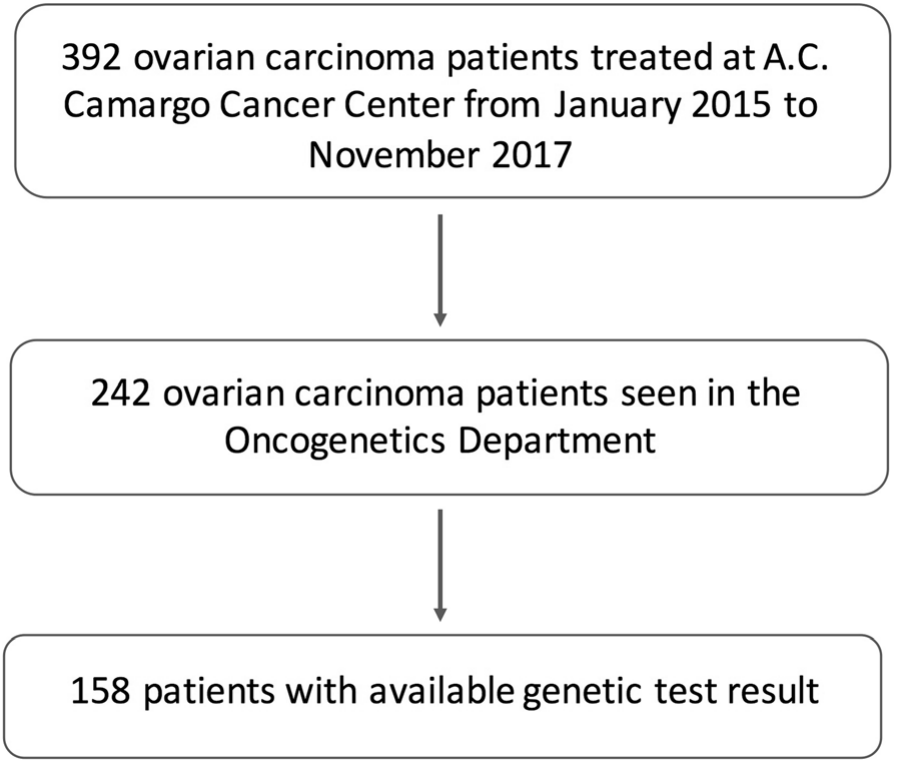
Frequency of pathogenic or likely pathogenic variants in *BRCA1* and *BRCA2* according to clinical characteristics

Patients with high grade serous carcinoma were found to carry a P or LP variant in 24.5% of cases compared to 13.0% for other histological subtypes (*p* = 0.105). Among histological subtypes other than HGSC, P or LP variants were found in one endometrioid tumor, one low grade serous carcinoma, two undifferentiated carcinomas and two carcinosarcomas. Patients with previous diagnosis of breast cancer were found to carry a P or LP variant in 8 of 12 cases (66.7%) compared to 18.0% for patients without previous history of breast cancer (*p* = 0.001). Patients with family history of ovarian or breast cancer in first or second degree relatives were found to carry a P or LP variant in 26.7% of cases compared to 16.9% for patients without previous history of family history of ovarian or breast cancer (*p* = 0.166). According to the time point genetic testing was taken, patients tested at the first line, platinum sensitive and platinum resistant settings were found to carry P or LP variants in 22.6, 21.2 and 7.1% of cases respectively (*p* = 0.493) (Fig. 2).

**Fig. 3:**
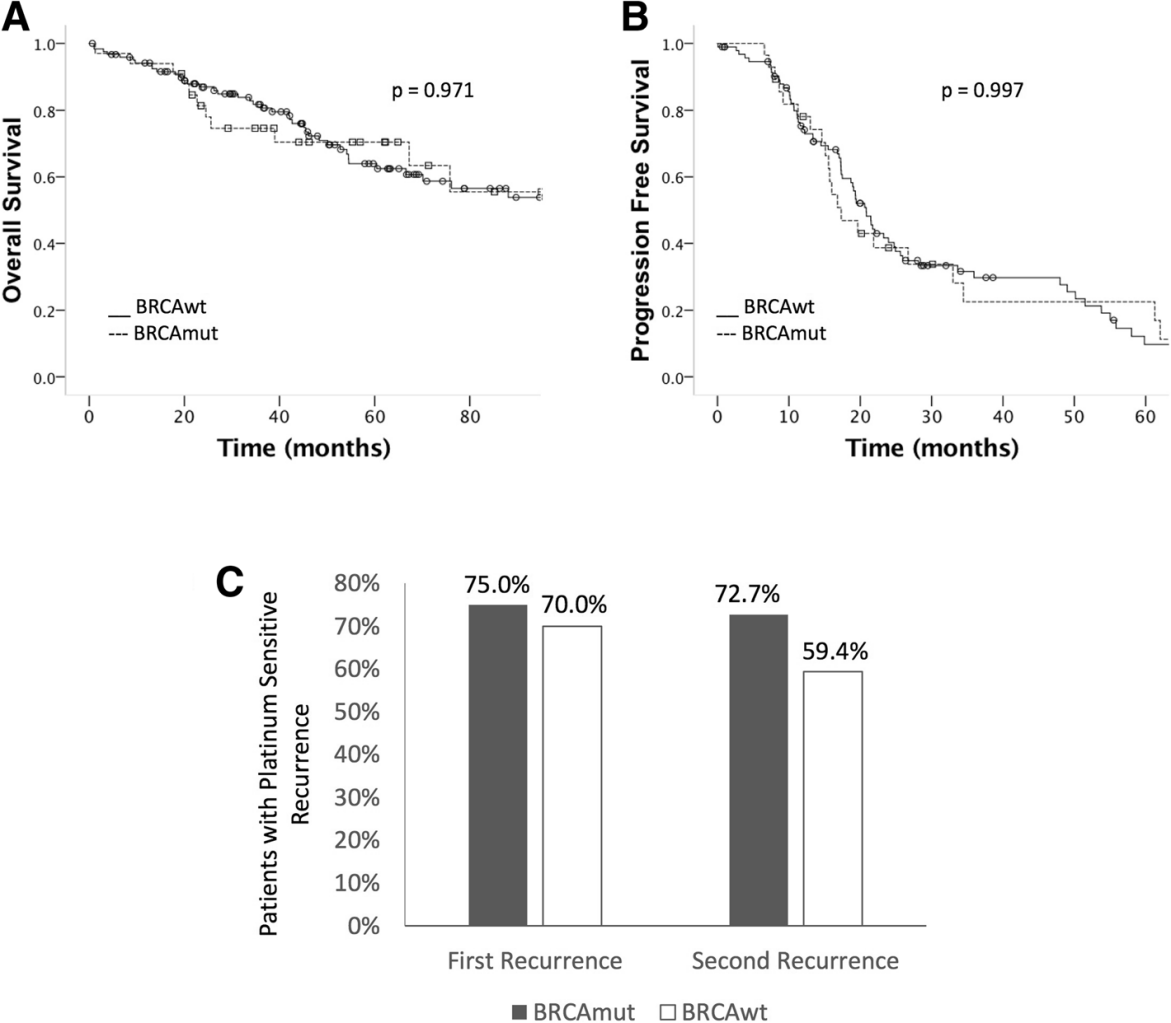
**a** Overall survival according to *BRCA* status, **b** Progression free survival according to *BRCA* status, **c** Frequency of platinum sensitive recurrence at first recurrence, and after second recurrence following the first platinum sensitive recurrence according to *BRCA* status. *BRCA*mut = pathogenic or likely pathogenic variant. *BRCA*wt = no pathogenic or likely pathogenic variants

Clinical endpoints indicating better prognosis and greater sensitivity to platinum therapy were not different according to P or LP carrier status. With a median follow-up time since diagnosis of 63.0 months, estimated median OS for all patients was 110.9 months and median PFS after first line treatment was 19.6 months. Comparing P and LP carriers with non-carriers, median OS was 122.8 months versus 110.9 months (*p* = 0.971) with a HR = 1.01 (95%CI 0.55–1.88; p = 0.971), and median PFS was 17.3 versus 20.8 months (*p* = 0.997) with a HR = 1.04 (95%CI 0.64–1.69; *p* = 0.889). First recurrence was classified as platinum sensitive in 75.0% versus 70.0% of cases (*p* = 0.876) and second recurrence after first platinum sensitive recurrence was classified as platinum sensitive in 72.7% versus 59.4% of cases (*p* = 0.494) (Fig. 3).

## Discussion

This is the largest series evaluating germline *BRCA1* and *BRCA2* with comprehensive gene analysis in a Brazilian population of ovarian cancer patients. We found a frequency of 17.1% of P and LP variants in *BRCA1,* 3.7% in *BRCA2*, and 3.7% of VUS considering both genes. Frequency and type of *BRCA* pathogenic variants among ovarian cancer patients varies according to the studied populations. Most European and US studies have shown frequencies between 11 and 17% [10–13, 15, 23]. One recent German study found a slightly higher frequency of *BRCA* pathogenic variants of 20% [30]. Studies from non-European or US populations showed higher frequencies, with 23% in Korean patients [21, 31], 27% in Chinese patients [19, 20] and 29% in Arabic patients [22]. Our findings put the frequency of pathogenic variants in *BRCA* in Brazilian ovarian patients on the upper frequency range of European and US studies. One previous study in Colombian patients found a frequency of 15% [14] and one previous Brazilian study found a frequency of 19% [25]. All these studies did comprehensive sequencing of *BRCA1* and *BRCA2.*

We included only patients that were referred to genetic counseling, this could lead to a selection bias because patients who are referred to genetic counseling could be those who have a more suspicious family history. Indeed, only 41% of our patients had no history of breast or ovarian cancer or personal history of breast cancer. This selection bias could be one of the reasons of the higher prevalence found in our study.

Three pathogenic variants in *BRCA1*, c.5266dupC (p.Gln1756Profs*74), c.3331_3334delCAAG (p.Gln1111Asnfs5*), and c.211A > G (p.Arg71Gly) were found more than once. The variant c.5266dupC (p.Gln1756Profs*74) is an Ashkenazi founder mutation that has already been described as a frequent variant in Brazilian patients with Hereditary Breast and Ovarian Cancer Syndrome [25, 32]. The variant c.3331_3334delCAAG (p.Gln1111Asnfs5*) is probably originated in Spain and has also been previously described as a frequent variant in ovarian cancer patients in Brazil and Latin America [25]. The variant c.211A > G (p.Arg71Gly) is a missense variant in the second-last position of the donor splice site of exon 5, which was proved to interfere in the splicing of this exon and results in a deletion of 22 bp of exon five, creating with the first bases of exon 6 a termination codon at position 64 [33]. It is a Spanish founder mutation and has been described in Latin American patients and, to a lesser extent, in Brazilian patients [33, 34].

One LP variant in *BRCA1* has not been described before c.4153_4154delCT (p.Leu1385Ilefs*5). It is a frameshift variant that leads to a premature stop codon and for these reason was classified as LP variant according to ACMG Guidelines [27].

Four variants in *BRCA1* and four variants in *BRCA2* had conflicting interpretation when reviewed at our institution in comparison to commercial laboratories reports, a frequency of 17.7% disagreement. Balmaña et al. reported discrepancies on variant interpretation among laboratories to be as high as 27% [35]. As expected, seven of the eight variants with conflicting interpretation were missense variants, and six of eight presented discordances between VUS and B or LB calls that would not have changed clinical management. Besides, 13% of patients did not have results on MLPA. The possibility of misinterpreted variants as non P or LP by commercial laboratories and the absence of MLPA results for 13% of patients may have led to an underestimation of *BRCA* P or LP variants in our study.

In accordance with previous studies, high frequency of *BRCA1* and *BRCA2* P or LP variants was present even in the subgroups with the lowest rates, for instance patients with no family history of breast or ovarian cancer and no previous breast cancer had P or LP variants in 16.9% of cases, justifying genetic testing for all patients [30]. The highest frequency was found among women with previous breast cancer, who had a chance of 66.7% of carrying P or LP variants in *BRCA1* or *BRCA2.* Patients with platinum sensitive relapse showed the same odds of carrying P or LP variants as patients after primary treatment, and patients with platinum resistant relapse showed P or LP variants in 7.1%. The smaller frequency of P and LP variants among patients tested at the platinum resistant scenario could be explained by the higher sensibility of *BRCA* mutated tumors to platinum therapy, and has also been described before [18]. We did not find a longer progression free or overall survival for P and LP carriers, but the small number of patients in the study and the number of deaths lower than expected for ovarian cancer patients hampers any conclusion on this regard. The small number of death events may be due to a not long enough follow-up time and the selection bias present in the study since patients diagnosed before 2015 were included if they were seen in the Oncogenetics department after 2015.

## Conclusion

In conclusion, we confirmed a frequency of P and LP variants in *BRCA1* and *BRCA2* in patients with ovarian carcinoma slightly above the expected for European and US populations but smaller than shown by Asian studies, demonstrating the specific genetic characteristics of Brazilian ovarian cancer patients who present a high frequency of the c.5266dupC (p.Gln1756Profs*74) variant and one not previously described variant.

### Acknowledgementes

We acknowledge Misses Gisleine Nieto for the support with the paperwork.

## Abbreviations

ACMG: American College of Medical Genetics
B: Benign
BRCA: Breast cancer
CC: Clear cell carcinoma
CS: Carcinossarcoma
D: Large deletion
E: Endometrioid carcinoma
F: Frameshift
FIGO: International Federation of Gynecology and Obstetrics
GCIG: Gynecological Cancer Intergroup
HBOC: Hereditary Breast and Ovarian Cancer Syndrome
HGSC: High grade serous carcinoma
IndDel: Inframe insertions or deletions
IQR: Interquartile range
LB: Likely benign
LGSC: Low grade serous carcinoma
LP: Likely pathogenic
M: Missense
MLPA: Multiplex Ligation-dependent Probe Amplification
N: Non sense
OS: Overall survival
P: Pathogenic
PFI: Platinum-free interval
PFS: Progression-free survival
RECIST: Response Evaluation Criteria in Solid Tumors
Ss: Splice site
UC: Undifferentiated carcinoma
USA: United States of America
VUS: Variant of unknown significance
